# Reduced lipolysis response to adipose afferent reflex involved in impaired activation of adrenoceptor-cAMP-PKA-hormone sensitive lipase pathway in obesity

**DOI:** 10.1038/srep34374

**Published:** 2016-10-03

**Authors:** Lei Ding, Feng Zhang, Ming-Xia Zhao, Xing-Sheng Ren, Qi Chen, Yue-Hua Li, Yu-Ming Kang, Guo-Qing Zhu

**Affiliations:** 1 Key Laboratory of Cardiovascular Disease and Molecular Intervention, Department of Physiology, Nanjing Medical University, Nanjing, Jiangsu 210029, China; 2 Department of Pathophysiology, Nanjing Medical University, Nanjing, Jiangsu 210029, China; 3 Department of Physiology and Pathophysiology, Cardiovascular Research Center, Xi’an Jiaotong University School of Medicine, Xi’an 710061, China

## Abstract

Chemical stimulation of white adipose tissue (WAT) causes adipose afferent reflex (AAR) and sympathetic activation. This study is to investigate the effects of AAR on lipolysis and the mechanisms of attenuated lipolysis response to enhanced AAR in obesity. Obesity was caused by high-fat diet for 12 weeks in rats. AAR was induced by injection of capsaicin into inguinal WAT or electrical stimulation of epididymal WAT afferent nerve. AAR caused sympathetic activation, which was enhanced in obesity rats. AAR increased cAMP levels and PKA activity, promoted hormone sensitive lipase (HSL) and perilipin phosphorylation, and increased lipolysis in WAT, which were attenuated in obesity rats. PKA activity, cAMP, perilipin and β-adrenoceptor levels were reduced, while HSL was upregulated in adipocytes from obesity rats. In primary adipocytes, isoproterenol increased cAMP levels and PKA activity, promoted HSL and perilipin phosphorylation, and increased lipolysis, which were attenuated in obesity rats. The attenuated effects of isoproterenol in adipocytes from obesity rats were prevented by a cAMP analogue dbcAMP. The results indicate that **r**educed lipolysis response to enhanced AAR in obesity is attributed to the impaired activation of β-adrenoceptor-cAMP-PKA-HSL pathway. Increased cAMP level in adipocytes rectifies the attenuated lipolysis in obesity.

It is well known that white adipose tissue (WAT) is innervated by the nerve including both sympathetic and sensory fibers^1^. Injection of leptin into the WAT increased sympathetic outflow to WAT, brown fat tissue (BAT), adrenal medulla, kidney, pancreas and liver, accompanied with the decreased vagal efferent activity to pancreas and liver^2^,^3^,^4^,^5^,^6^. We found that chemical stimulation of WAT with bradykinin, adenosine or capsaicin caused a similar sympathetic activation and a mild pressor response in normal rats, and the sympatho-excitatory reflex was called adipose afferent reflex (AAR)^5^. The AAR was mediated by ionotropic glutamate receptors and modulated by superoxide anions and melanocortin 4 receptors in paraventricular nucleus (PVN) of hypothalamus^7^,^8^,^9^. The interaction of the WAT and brain may be important in promoting lipolysis and energy expenditure, and is proposed to be helpful for keeping total body fat and body weight stable and preventing obesity via its reflex control of sympathetic outflow^1^,^10^,^11^,^12^. However, there is still no direct evidence that AAR promotes lipolysis.

Obesity is associated with increased incidence of cardiovascular disease^13^. Sympathetic overdrive is present in obese patients and contributes to hypertension and other cardiovascular diseases^14^,^15^,^16^. We recently found that the AAR was enhanced in high-fat diet (HFD)-induced obese rats, which greatly contributes to the excessive sympathetic activation and hypertension in obesity^17^. Sympathetic activity is known to increase lipolysis via adrenergic receptor-medicated activation of hormone sensitive lipase (HSL)^1^,^18^. However, it is unknown whether the enhanced AAR induces lipolysis. A more interesting question is why enhanced AAR and sympathetic outflow in obese rats fails to prevent obesity. The present study is designed to investigate the effects of AAR on lipolysis and the mechanisms of the attenuated lipolysis response to the enhanced AAR in obese rats. On the other hand, visceral adipose tissue (VAT) and subcutaneous adipose tissue (SAT) largely differ concerning their metabolic characteristics^19^. Therefore, the difference between VAT and SAT in the AAR-induced lipolysis was compared in the present study.

## Results

### Effects of capsaicin-induced AAR on sympathetic activation and lipolysis

**Figure 1:**
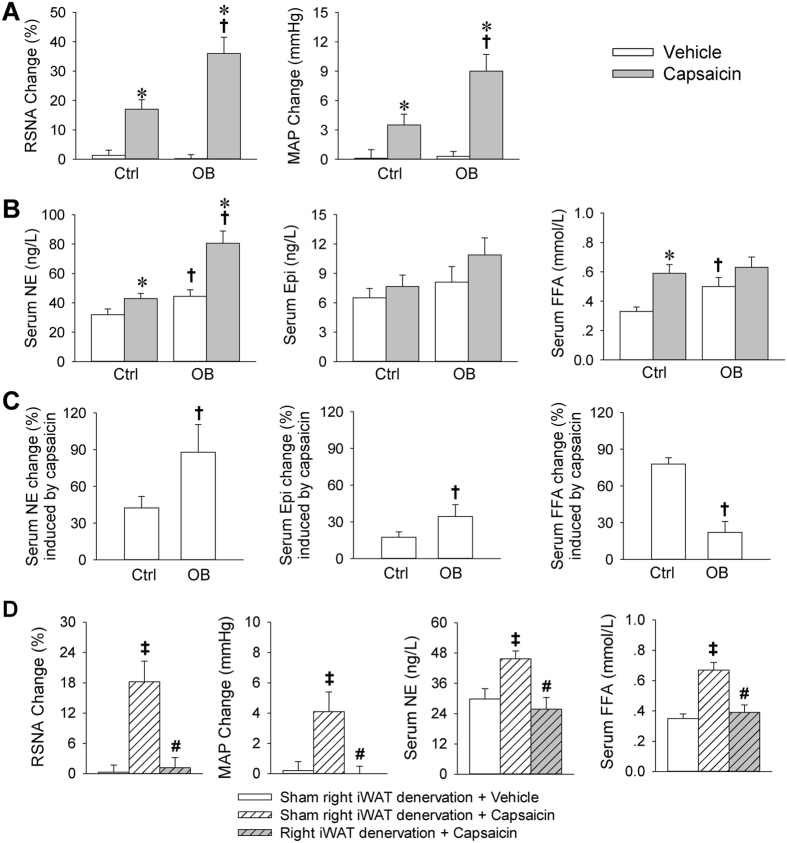
Sympathetic activation and lipolysis effects of capsaicin-induced AAR in Ctrl and OB rats. (**A**) Effects of iWAT infusion of capsaicin on RSNA and MAP change. (**B**) Effects of iWAT infusion of capsaicin on serum NE, epinephrine (Epi) and FFA levels. (**C**) Percentage change of serum NE, Epi and FFA induced by the iWAT infusion of capsaicin. (**D**) Right iWAT surgical denervation abolished the capsaicin-induced sympathetic activation and lipolysis in normal rats. Values are mean ± S.E.M. *P < 0.05 vs. Vehicle; ^†^P < 0.05 vs. Ctrl; ^‡^P < 0.05 vs. Sham right iWAT denervation + Vehicle; ^#^P < 0.05 vs. Sham right iWAT denervation + capsaicin. n = 6 for each group.

Body weight was much greater in obesity (OB) rats than that in control rats (Table S1). Right inguinal WAT (iWAT) injection of capsaicin caused greater increases in renal sympathetic nerve activity (RSNA) and mean arterial pressure (MAP) in OB rats than those in control (Ctrl) rats (Fig. 1A). Serum norepinephrine (NE) and free fatty acids (FFA) levels were increased in OB rats. Capsaicin increased serum NE level in both Ctrl and OB rats. It increased serum FFA level in Ctrl rats, but only induced a tendency in increasing serum FFA in OB rats (Fig. 1B). The capsaicin-induced increase in serum NE and epinephrine (Epi) in OB rats was much greater than those in Ctrl rats (NE: +87.9 ± 14.9 vs. +42.4 ± 9.4%; Epi: +34.4 ± 9.6 vs. +17.5 ± 4.2%), while the increase in serum FFA in OB rats was much less than that in Ctrl rats (+22.2 ± 5.0 vs. +77.9 ± 5.2%) (Fig. 1C). The effects of capsaicin in right iWAT in normal rats were abolished by the surgical right iWAT denervation (Fig. 1D). Our previous study showed that intravenous injection of capsaicin or injection of same dose of capsaicin into the adjacent skeletal muscle or skin had no significant effect in inducing AAR^5^. Therefore, the capsaicin-induced effects were caused by increased iWAT inputs rather than the direct effects of capsaicin in the iWAT or the effects of capsaicin diffusion to adjacent tissues. Furthermore, primary SAT adipocytes derived from normal rats were used to determine whether capsaicin has a direct effect on lipolysis in adipocytes. Although activation of β-adrenergic receptors with isoproterenol (ISO) greatly increased glycerol and FFA release at both 1 h and 24 h, capsaicin only caused a mild glycerol and FFA release at 24 h and failed to cause significant glycerol and FFA release at 1 h in adipocytes (Figure S1). The results suggest that the lipolysis effect induced by injection of capsaicin into iWAT is attributed to sympathetic activation due to the AAR rather than its direct effect on lipolysis. On the other hand, injection of capsaicin into VAT caused greater increases in RSNA and MAP, but less increase in FFA release in OB rats than those in Ctrl rats (Figure S2), which were similar to the effects induced by injection of capsaicin into SAT.

### Effects of electrical stimulation-induced AAR on sympathetic activation and lipolysis

**Figure 2:**
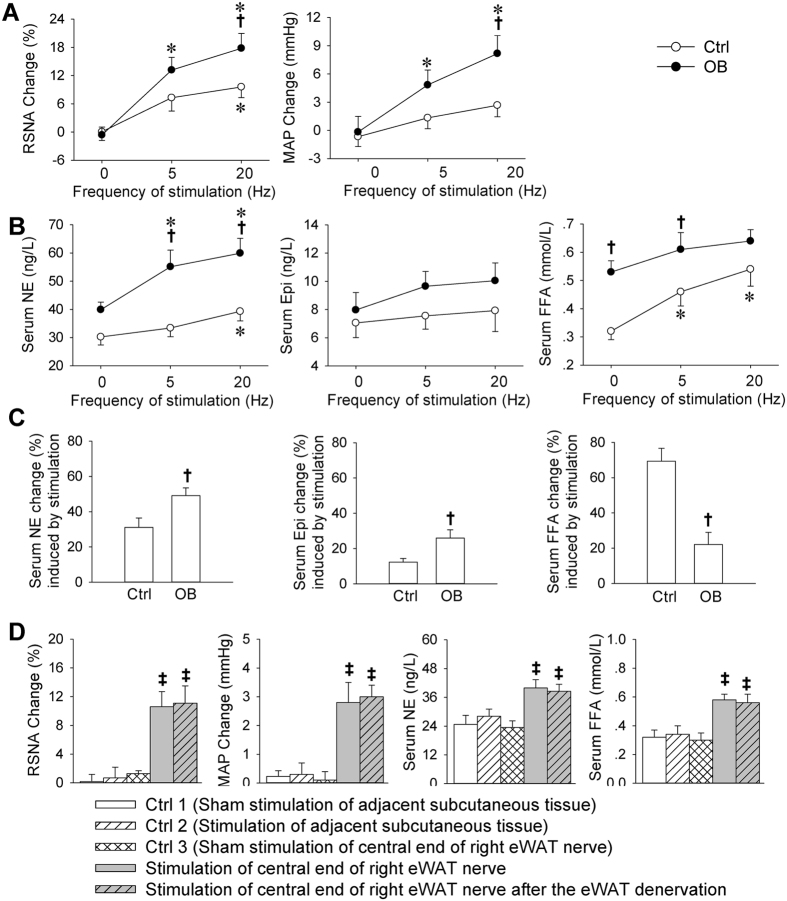
Sympathetic activation and lipolysis effects of electrical stimulation-induced AAR in Ctrl and OB rats. (**A**) Effects of stimulation of eWAT afferent nerve on RSNA and MAP change. (**B**) Effects of stimulation of eWAT afferent nerve on serum NE, Epi and FFA levels. (**C**) Percentage change of serum NE, Epi and FFA induced by the stimulation of WAT afferent nerve. (**D**) Stimulation of central end of eWAT nerve induced sympathetic activation and lipolysis while stimulation of adjacent subcutaneous tissue failed to induce sympathetic activation and lipolysis in normal rats. Values are mean ± S.E.M. *P < 0.05 vs. 0 Hz; ^†^P < 0.05 vs. Ctrl. ^‡^P < 0.05 vs. Ctrl 1, Ctrl 2 or Ctrl 3. ^#^P < 0.05 vs. Stimulation of central end of right eWAT nerve. n = 6 for each group.

Electrical stimulation of right epididymal WAT (eWAT) nerve caused greater increases in RSNA and MAP in OB rats than those in Ctrl rats (Fig. 2A). The electrical stimulation increased serum NE level in both Ctrl and OB rats. However, it induced a significant increase in serum FFA only in Ctrl rats (Fig. 2B). The stimulation-induced increases in serum NE and Epi in OB rats were more than those in Ctrl rats, while the increase in serum FFA in OB rats was much less than that in Ctrl rats (Fig. 2C). Furthermore, electrical stimulation of adjacent subcutaneous tissue in normal rats failed to cause sympathetic activation and lipolysis, and right eWAT denervation had no significant effect on the AAR induced by electrical stimulation of central end of the right eWAT nerve, excluding the possibility that the effects may be caused by the spreading stimulation to other tissues (Fig. 2D).

### Effects of capsaicin-induced AAR on HSL, perilipin, ATGL, cAMP and PKA

**Figure 3:**
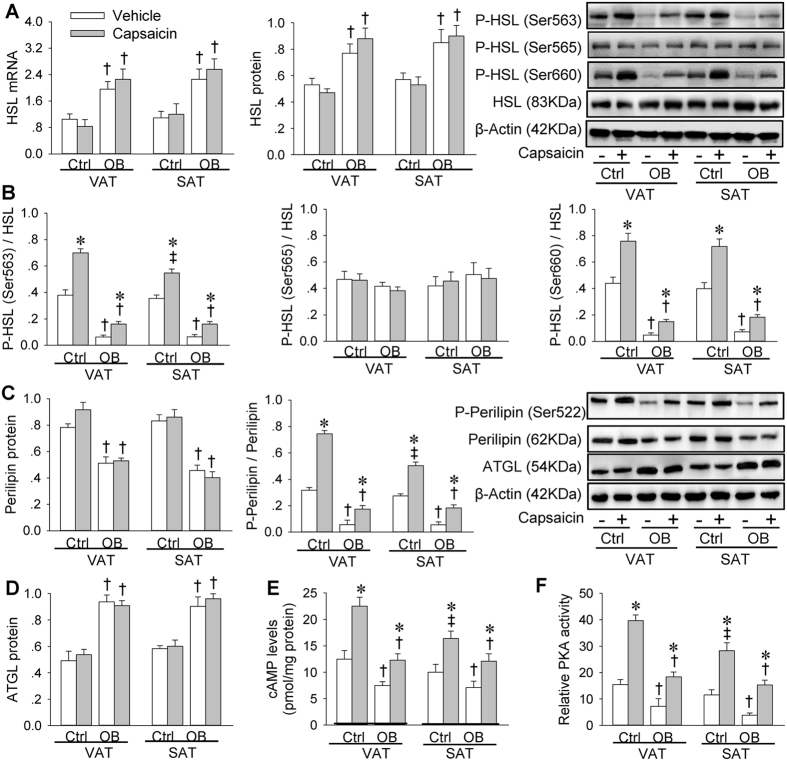
HSL, perilipin, ATGL, cAMP and PKA in VAT and SAT in Ctrl and OB rats treated with stimulation of adipose afferents with capsaicin. (**A**) Relative HSL expression. (**B**) HSL phosphorylation. (**C**) Perilipin expression and phosphorylation. (**D**) ATGL expression. (**E**) cAMP levels. (**F**) PKA activity. Values are mean ± S.E.M. *P < 0.05 vs. Vehicle; ^†^P < 0.05 vs. Ctrl; ^‡^P < 0.05 vs. VAT. n = 4 for each group in (**A**–**D**), and n = 6 for each group in (**E**,**F**).

HSL mRNA and protein were upregulated in OB rats. There were no significant difference in HSL expression between VAT and SAT in both Ctrl and OB rats. Capsaicin-induced AAR had no significant effect on the HSL expressions (Fig. 3A). Phosphorylation of HSL at Ser660 and Ser563 in both VAT and SAT were reduced in OB rats. Capsaicin-induced AAR promoted HSL phosphorylation at Ser660 and Ser563 in both VAT and SAT, but the effect of AAR was greatly reduced in OB rats compared with those in Ctrl rats. Capsaicin-induced AAR caused a greater increase in the HSL phosphorylation at Ser563 in VAT than that in SAT in Ctrl rats, but not in OB rats (Fig. 3B). Perilipin was downregulated, but adipose triglyceride lipase (ATGL) was upregulated in OB rats (Fig. 3C,D). Perilipin phosphorylation levels were lower in OB rats than those in Ctrl rats. Capsaicin-induced AAR promoted perilipin phosphorylation in both VAT and SAT, but the effect of AAR was smaller in OB rats than that in Ctrl rats. Capsaicin-induced AAR caused a greater increase in perilipin phosphorylation level in VAT than that in SAT in Ctrl rats, but not in OB rats (Fig. 3C). Intracellular cAMP levels and PKA activity were decreased in OB rats. Capsaicin-induced AAR increased cAMP levels and PKA activity in both VAT and SAT, but the effect of AAR was smaller in OB rats than that in Ctrl rats. Capsaicin-induced AAR caused greater increases in cAMP levels and PKA activity in VAT than that in SAT in Ctrl rats, but not in OB rats (Fig. 3E,F).

### Adrenergic receptor expression in VAT and SAT

**Figure 4:**
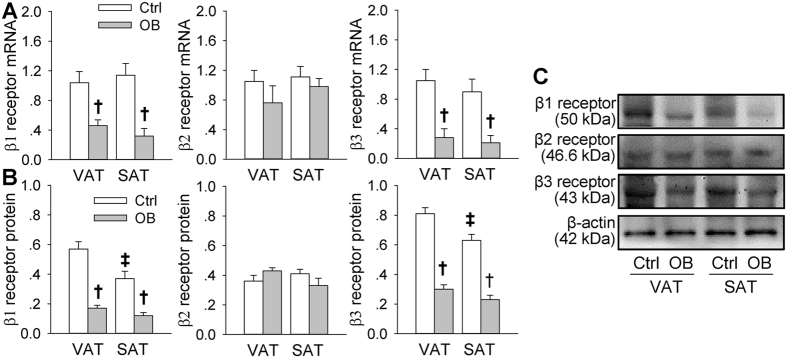
Adrenergic receptor mRNA and β-adrenergic receptor protein in VAT and SAT of Ctrl and OB rats. (**A**) Relative values of β_1_, β_2_ and β_3_ receptor mRNA. (**B**) Relative values of β_1_, β_2_ and β_3_ receptor protein. (**C**) Representative images of Western blot showing β_1_, β_2_ and β_3_ receptor protein. Values are mean ± S.E.M. *P < 0.05 vs. Vehicle; ^†^P < 0.05 vs. Ctrl; ^‡^P < 0.05 vs. VAT. n = 4 for each group.

Adrenergic β_1_ and β_3_ receptor mRNA and protein expression in both VAT and SAT were down-regulated in OB rats. The β_1_ and β_3_ receptor protein levels in Ctrl rats were lower in SAT than that in VAT, but not in OB rats. There was no significant difference in the β_2_ receptor mRNA and protein expression between VAT and SAT or between Ctrl and OB rats (Fig. 4A–C).

### Effects of β-adrenergic receptor agonist ISO on lipolysis in primary VAT and SAT adipocytes

**Figure 5:**
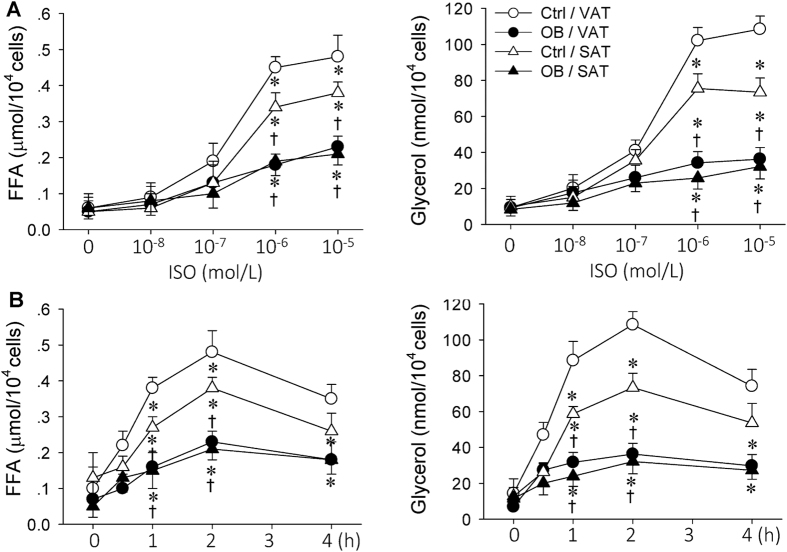
Comparison of the effects of β-receptor agonist ISO on lipolysis in Ctrl and OB rats. (**A**) Dose effect of ISO in primary VAT and SAT adipocytes. (**B**) Time effect of ISO in primary VAT and SAT adipocytes. Values are mean ± S.E.M. *P < 0.05, vs. Ctr/VAT; ^†^P < 0.05, vs. Ctr/SAT. n = 6 for each group.

Capsaicin-induced AAR caused a greater increase in NE contents in adipose tissues of OB rats than that of Ctrl rats, but had no significant effect on Epi contents in adipose tissues of both Ctrl and OB rats, suggesting capsaicin-induced AAR causes NE release from sympathetic nerves innervating WAT (Figure S3). ISO and NE caused similar lipolysis effect in adipocytes derived from VAT and SAT in Ctrl and OB rats (Figure S4). It is well known that catecholamine plays a central role in promoting lipolysis in WAT via β-adrenergic receptors^20^. ISO selectively activates β-adrenergic receptors, but NE can activate both α- and β-adrenergic receptors. ISO is widely used to test lipolysis in WAT. Thus, ISO was used to mimic the effects of AAR-induced sympathetic activation on adipocytes *in vivo* in the present study. ISO stimulated lipolysis of primary VAT and SAT adipocytes derived from both Ctrl and OB rats. ISO-induced lipolysis effect was greatly reduced in VAT and SAT adipocytes from OB rats compared those from Ctrl rats. The lipolysis effect was greater in VAT adipocytes than that in SAT adipocytes from Ctrl rat. However, no significant difference in lipolysis was found between the VAT and SAT adipocytes originated from OB rats. Furthermore, the maximal lipolysis effect was observed 2 h after incubation with ISO, and 10^−6^ mol/L of ISO almost reached its maximal lipolysis effect (Fig. 5A,B). Therefore, 10^−6^ mol/L of ISO incubated for 2 h was selected to stimulate lipolysis of the primary adipocytes in the following studies.

### Effects of ISO on cAMP levels and PKA activity in primary VAT and SAT adipocytes

**Figure 6:**
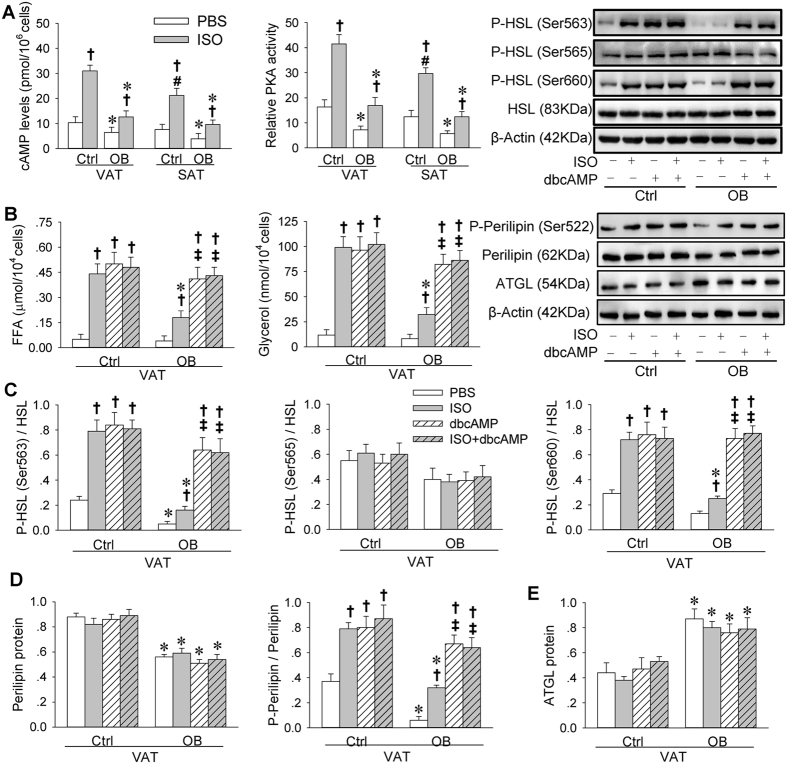
cAMP, PKA, lipolysis, HSL, perilipin and ATGL in primary VAT and SAT adipocytes of Ctrl and OB rats. (**A**) Effects of ISO on cAMP levels and relative PKA activity. (**B**) Effects of ISO and dbcAMP on lipolysis. (**C**) Effects of ISO and dbcAMP on HSL phosphorylation. (**D**) Effects of ISO and dbcAMP on perilipin phosphorylation. (**E**) Effects of ISO and dbcAMP on ATGL expression. The measurements were carried out after 2-h of incubation with BPS, ISO (10^−6^ mol/L), dbcAMP (10^−3^ mol/L) or ISO + dbcAMP. Values are mean ± S.E.M. *P < 0.05 vs. Ctrl; ^†^P < 0.05 vs. PBS; ^‡^P < 0.05 vs. ISO; ^#^P < 0.05 vs. VAT. n = 6 for each group in (**A**,**B**); n = 4 for each group in (**C**–**E**).

The cAMP levels and PKA activity were lower in VAT and SAT adipocytes from OB rats than those from Ctrl rats. ISO increased cAMP levels and PKA activity, but the effects of ISO were much less in VAT and SAT adipocytes from OB rats than those from Ctrl rats. Moreover, the ISO-induced increase in cAMP levels and PKA activity were less in SAT adipocytes than that in VAT adipocytes from Ctrl rats (Fig. 6A).

### Effects of dbcAMP on lipolysis in primary VAT adipocytes

A cAMP analogue dbutyryl cyclic AMP (dbcAMP) was used to rectify the insufficient cAMP in the VAT adipocytes from OB rats. Although the ISO-induced lipolysis effect was greatly reduced in the adipocytes from OB rats compared with those from Ctrl rats, dbcAMP in the adipocytes from OB rats induced a similar degree of lipolysis effect to the ISO- or dbcAMP-induced effect in the adipocytes from Ctrl rats. Combined incubation of ISO and dbcAMP failed to cause greater lipolysis effect than dbcAMP alone (Fig. 6B).

### Effects of dbcAMP on HSL, perilipin and ATGL in primary VAT adipocytes

The basal phosphorylated HSL (at Ser563 and Ser660) levels were very low in the adipocytes from OB rats compared with those from Ctrl rats. The ISO-induced HSL phosphorylation effect was much weaker in the adipocytes from OB rats than those from Ctrl rats. The dbcAMP in the adipocytes from OB rats induced a similar degree of HSL phosphorylation effect to the ISO- or dbcAMP-induced effect in the adipocytes from Ctrl rats. Combined incubation of ISO and dbcAMP failed to cause greater HSL phosphorylation effect than dbcAMP alone (Fig. 6C). Perilipin was downregulated, but ATGL was upregulated in adipocytes from OB rats (Fig. 6D,E). Perilipin phosphorylation levels were lower in OB rats than those in Ctrl rats. ISO promoted perilipin phosphorylation, but the effect was smaller in OB rats than that in Ctrl rats. A cAMP analogue dbcAMP in the adipocytes from OB rats induced a similar degree of perilipin phosphorylation effect to the ISO- or dbcAMP-induced effect in the adipocytes from Ctrl rats. Combined incubation of ISO and dbcAMP failed to cause greater perilipin phosphorylation effect than dbcAMP alone (Fig. 6D).

## Discussion

Sympathetic activation is a crucial factor for lipolysis in the WAT via NE-mediated β-adrenoceptor stimulation^1^. The AAR induced by chemical stimulation of WAT causes a general sympathetic activation^5^,^11^, and the interaction between brain and WAT is likely involved in the control of lipolysis realized by regulating sympathetic activity^21^. In the present study, we found that chemical stimulation of right iWAT sensory afferents with capsaicin increased serum FFA levels in normal rats, which was abolished by cutting the iWAT nerves. The results were confirmed by the findings that electrical stimulation of right eWAT afferent fibers also increased serum FFA levels in normal rats. These findings firstly provide a direct evidence that the AAR induced by WAT stimulation promotes lipolysis via sympathetic activation in normal rats. This reflex may be involved in keeping the total body fat mass and body weight stable in physiological situation.

Sympathetic outflow is known to be increased in obese patients^14^,^15^,^16^. We found that the AAR was enhanced in HFD-induced obese rats, and the enhanced AAR contributed to the sympathetic activation and hypertension in obesity^17^. In the present study, we found that the enhanced AAR in obese rats caused a major increase in sympathetic activity, but only a minor increase in serum FFA, which was different with normal control rats that the AAR induced a slight increase in sympathetic activity but a great increase in serum FFA. The results were further confirmed by the finding that the lipolysis effect caused by activating β-adrenergic receptor with ISO was weaker in primary adipocytes from obese rats than that from control rats. These results indicate that the WAT sensitivity to the AAR-induced sympathetic activation is greatly reduced in obese rats. The reduced sensitivity of WAT in obese rats may be one of important causes that enhanced AAR failed to prevent obesity.

It is well known that sympathetic activity promotes lipolysis in adipocytes^1^,^18^. Activation of β-adrenergic receptors increases intracellular cAMP contents, and cAMP activates PKA which activates HSL via promoting its phosphorylation^22^,^23^. HSL is important for the degradation of triacylglycerol in adipose tissues. Ser563, Ser659 and Ser660 of HSL are the major phosphorylation sites related to the HSL activity, although Ser563 may not affect HSL activity directly^24^,^25^. HSL phosphorylation at Ser563 or Ser660 activates HSL and lipolysis, while phosphorylation at HSL Ser565 prevents HSL activation^26^. In the absence of β-adrenoceptor stimulation, perilipin is mainly located on the surface of lipid droplet to prevent lipases from accessing lipid, whereas HSL is in cytoplasm. Upon β-adrenoceptor stimulation, activated PKA phosphorylates perilipin and HSL^22^. HSL translocates to the lipid droplet leading to enhancement in lipolysis^27^. Adipose triglyceride lipase (ATGL) functions essentially as a triacylglycerol lipase, ATGL upregulation promotes triacylglycerol breakdown^28^,^29^. We found that the cAMP levels, PKA and HSL activity, and perilipin phosphorylation in WAT were reduced in obese rats compared with control rats. Capsaicin-induced AAR increased cAMP levels, PKA and HSL activity, and perilipin phosphorylation in the WAT, and the effects were much weaker in obese rats than those in control rats. The cAMP levels and PKA activity were lower in adipocytes from obese rats than those from control rats. ISO-induced increases in HSL activity, cAMP levels and PKA activity, and perilipin phosphorylation were less in adipocytes from obese rats than those from control rats. A cAMP analogue dbcAMP normalized the reduced lipolysis and HSL activation, and perilipin phosphorylation effects in the adipocytes from obese rats, while ISO plus dbcAMP failed to cause greater effect than dbcAMP alone. These results indicate that the decreased cAMP production and PKA activation in the WAT reduces the HSL activation, which contributes to the attenuated lipolysis in WAT in obesity. On the other hand, upregulation of HSL and AGTL and downregulation of perilipin in obesity rats may attribute to compensatory effects to reduced lipolysis.

There are three β-adrenoceptor subtypes (β_1_, β_2_ and β_3_). The β_1_ and β_2_ receptors are expressed in many body tissues including adipocytes, and the β_3_ receptors are found in several human fat depots, but predominantly in adipocytes in rodents. Each of β-adrenoceptor subtypes is coupled to Gα subunit of Gs protein, and stimulation of these receptors increases in intracellular cAMP levels and causes lipolysis^30^. We found that the β_1_ and β_3_ receptor mRNA and protein were down-regulated in rats with HFD-induced obesity, which may partially contribute to the reduced cAMP production, PKA and HSL activation responses to the enhanced AAR and sympathetic outflow in obesity.

Adipose tissue plays an essential role in regulating energy balance through its metabolic, cellular and endocrine functions^31^,^32^. Metabolic remodeling of WAT has been found in obesity^33^. WAT is generally divided into SAT and VAT. Sympathetic activity was more closely associated with the level of abdominal VAT than abdominal SAT or total fat mass in obese humans^34^. Visceral obesity correlates with increased risk of insulin resistance and cardiovascular diseases, while increase of subcutaneous fat is associated with favorable plasma lipid profiles^35^,^36^. We found that ISO-induced lipolysis effect was greater in VAT adipocytes than that in SAT adipocytes from normal control rats, while the lipolysis effect was inhibited and no significant difference in the lipolysis effect was found between the VAT adipocytes and the SAT adipocytes from obese rats. These results suggest that the AAR-induced sympathetic activation predominantly reduced the VAT mass rather than SAT mass in physiological situation. The disappearance of the priority role may result in an excessive increase in VAT in obesity, which is adverse to the body. It was found that the β_1_ and β_3_ receptor expression in VAT was higher than that in SAT, and that the AAR-induced increases in the cAMP level, PKA activity, HSL phosphorylation at Ser563, and perilipin phosphorylation at Ser552 in VAT were more than those in SAT in Ctrl rats, which may be responsible for the reduced lipolysis response in SAT compared with VAT in normal rats. The reduced β_1_ and β_3_ receptor expression, and the attenuated cAMP and HSL phosphorylation responses to AAR in both VAT and SAT may be responsible for the reduced lipolysis response in obese rats.

It is known that chemical stimulation of WAT causes a general activation of sympathetic nervous system^2^,^3^,^4^,^5^,^6^. Our previous study showed that capsaicin-induced AAR increased the WAT sympathetic nerve activity, which was similar to the increase in the RSNA^5^. In the present study, we recorded the RSNA as a marker for the sympathetic activation instead of the WAT sympathetic nerve activity. The RSNA may be better in the evaluation of the general sympathetic activation than the WAT sympathetic nerve activity of a local fat pad. In strengthening the marker of the sympathetic activation, serum NE levels were used as an index of the AAR-induced change in sympathetic activity. Excessive sympathetic activity is known to play a crucial role in the pathogenesis of hypertension^37^,^38^,^39^,^40^. The enhanced AAR and sympathetic activity may be a mechanism to compensate the attenuated lipolysis in obesity. However, the excessive activation of AAR and sympathetic activity actually induces hypertension and other adverse effects. We expect that the improvement of the WAT sensitivity to sympathetic activity to promote lipolysis will interrupt the enhanced AAR-related vicious circle in obesity, and thereby prevent the excessively sympathetic activation and hypertension.

## Materials and Methods

### Rat obesity models

Male Sprague-Dawley rats (n = 90) weighing between 300–350 g were randomly divided into two groups, which respectively received a control diet (Ctrl, 12% kcal as fat, n = 30) and a high-fat diet (HFD, 42% kcal as fat, n = 60) to induce obesity models as we previously reported^17^. After 12 weeks, rats were ranked based on their body weight gain. The HFD rats with a greater weight gain than the Ctrl rat with the greatest weight gain were defined as obese rats (OB)^41^,^42^. All rats were housed in a temperature and humidity-controlled room with a 12-hour light/dark cycle with free access to food and water. The experimental procedures were approved by the Experimental Animal Care and Use Committee of Nanjing Medical University and complied with the Guide for the Care and Use of Laboratory Animals (NIH publication, 8th edition, 2011). All procedures performed in studies involving animals were in accordance with the ethical standards of the institution or practice at which the studies were conducted.

### General experimental procedures *in vivo*

Rats were anesthetized with intraperitoneal injection of urethane (800 mg/kg) and α-chloralose (40 mg/kg). The depth of anesthesia was determined by the absence of corneal reflexes and paw withdrawal response to a noxious pinch. The rat was mechanically ventilated using a rodent ventilator (model 683, Harved Apparatus Inc, USA). The right carotid artery was cannulated for continuous recording of blood pressure. Left renal nerve was isolated through a retroperitoneal incision and was cut distally to eliminate its afferent activity. RSNA and MAP were simultaneously recorded by a PowerLab data acquisition system (8/35, AD Instruments, Castle Hill, Australia) as we reported previously^43^.

### Capsaicin-induced AAR

Low concentration of capsaicin is known to causes neuronal excitation without damage of the sensory neurons, and is commonly used to determine the function of sensory afferents^44^,^45^,^46^. In the present study, the AAR was induced by injection of low concentration of capsaicin into the right iWAT as we previously reported^5^,^17^. Briefly, right iWAT was exposed through an inguinal area incision. Four stainless steel tubes (0.31 mm outer diameter) connected with a 4-channel programmable pressure injector (PM2000B, MicroData Instrument, NJ, USA) were inserted into the iWAT. The tube tips were 4 mm apart from each other, and 3 mm below the surface of the fat pads. The AAR were induced by simultaneous injections of capsaicin into the four sites of right iWAT (1.0 nmol/μl, 4.0 μl/min for each site, lasting for 2 min). This dose of capsaicin was selected according to the dose-effects relationship in our previous study^5^. At the end of the experiment, the same volume of Evans blue was injected into the iWAT. Histological identification showed the dye was localized in the injection sites of the iWAT and the diameter of diffusion was less than 3 mm.

### Electrical stimulation-induced AAR

The AAR induced by electrical stimulation of the right eWAT nerve was conducted for further confirming the results from the capsaicin-induced AAR. A midline incision was made to expose right eWAT, and a drop of 1% toluidine blue was applied to the fat pad to facilitate visualization of the eWAT nerve. The nerve was isolated and cut distally. A pair of silver stimulating electrodes was placed on the central end of this nerve. Stimulus was delivered with a stimulator (model S88, Grass Instruments, Quincy, MA, USA) and a stimulus isolation unit. The different frequencies of the stimulus (0, 5 or 20 Hz) were randomly delivered. The stimulation lasted 10 min for each stimulation period. The interval between each stimulation period was at least 60 min for complete recovery and the stimulation was up to three times for each animal. The voltage of the stimulus was kept at 10 V and a pulse width was 1 ms^47^.

### Evaluation of AAR

AAR was evaluated by the responses of RSNA, MAP, NE and FFA levels to the injection of capsaicin into the iWAT or the electrical stimulation of the eWAT nerve. The RSNA and serum NE level were used as the markers of sympathetic activation, and the serum FFA level was used as a marker of lipolysis.

### Real-time quantitative PCR

The rat was anaesthetized with an overdose of sodium pentobarbital (150 mg/kg, iv). The adipose tissues were quickly removed and frozen with liquid nitrogen and stored at −80 °C. Total RNA was isolated from tissues using Trizol reagent (Invitrogen, CA, USA). The HSL, β_1_ receptor, β_2_ receptor and β_3_ receptor mRNA levels in the WAT were determined with Real-time quantitative PCR using a StepOnePlus Real-Time PCR System (Applied Biosystems, Foster City, CA, USA). All genes expression levels were normalized by β-actin levels. The sequences of primers were listed in the table (Table S2).

### Western blot

HSL phosphorylation and adrenergic receptor protein expression were determined with Western blot method. Briefly, after electrophoresis and transmembrane processes, the membranes were blocked with 5% non-fat milk in TBS-T buffer, and incubated with antibodies targeting phosphorylated HSL (Ser-563, Ser-565 or Ser-660), total HSL, β_1_ receptor, β_2_ receptor, β_3_ receptor or β-actin, respectively. β-actin was used as a loading control. Band intensity was quantified with Image J software (National Institutes of Health, Bethesda, MD, USA).

### Measurement of NE, Epi, cAMP and PKA

Commercial ELISA kits were used to measure NE and epinephrine levels (Cloud-Clone Corp. Houston, TX, USA) and cAMP levels (R&D Systems, Inc. Minneapolis, MN, USA). Standard diluent buffer or samples diluent were added and incubated in the appropriate well of specific antibody pre-coated microtiter plate. Conjugate was added and then washed. Stop solution were added into the appropriate well and the final solution was read at 450 nm for the NE and the Epi, and 540 nm for cAMP using a microplate reader (ELX800, BioTek, Vermont, USA). PKA activity was measured with PKA Kinase Assay Kits (ImmuneChem Pharmaceuticals Inc, Burnaby, British Columbia, Canada).

### Isolation of primary adipocytes

Perirenal adipose depots and abdominal subcutaneous adipose tissues were used as a representative of VAT and SAT respectively^48^,^49^. Adipocytes were isolated as previously described^50^. Briefly, fat pads were harvested under sterile conditions and minced thoroughly (~2–3 mm pieces in diameter). Minced fat tissue (~2 g) were digested at 37 °C for 1 h in 15 ml of Krebs-Ringer bicarbonate buffer (KRB, pH 7.4) containing glucose (5.5 mM), 4% fatty acid-free bovine serum albumin (BSA) and collagenase (1 mg/ml). The digested tissue was filtered through a nylon mesh and fat cells were collected in a 50-ml plastic tube, and then was consecutively washed with KRB buffer three times. The adipocytes were preincubated in phenol red-free and serum-free DMEM containing 2% defatted bovine serum albumin at 37 °C for 1 h before treatments. Then, adipocytes were incubated in the presence or absence of the tested agents, followed by measurements^51^. Adipocyte number was determined according to previously described method^52^.

### Lipolysis assays

Lipolysis was determined by assaying FFA and glycerol levels^53^. Commercial acyl-CoA oxidase-based colorimetric kits (Wako Chemicals, Richmond, VA) were used for measuring FFA levels in the medium^54^. The Free Glycerol Determination Kits (Sigma Aldrich, St. Louis, MO) were used for determining the glycerol levels in the medium. The coupled enzyme reactions of this assay produced quinoneimine dye that was determined at 540 nm. The increase in absorbance at 540 nm is directly proportional to the free glycerol concentration of the sample^55^.

### Chemicals

Dibutyryl cyclic AMP (dbcAMP), isoproterenol (ISO) and capsaicin were obtained from Sigma Chemical Co. (St. Louis, MO, USA). Fatty acid-free bovine serum albumin (BSA) was purchased from equitech-bio, Inc. (Kerrville, TX, USA). Antibodies of HSL and their phosphorylated forms were purchased from Cell Signaling Technology Inc. (Danver, MA, USA). Antibodies of β_1_ receptor, β_2_ receptor, β_3_ receptor, ATGL and β-Actin were obtained from Abcam (Cambridge, UK). Antibodies of perilipin was purchased from Vala sciences (San Diego, CA, USA). Capsaicin stock solution was dissolved in absolute ethanol and was diluted before injection to a final concentration of 1% of the stock solution, 1% of Tween 80, and 98% of normal saline. Vehicle was used as control.

### Statistical analysis

Comparisons between two groups were made by Student’s t test. One-way or two-way ANOVA followed by post hoc Bonferroni test was used when multiple comparisons were made. All data were expressed as mean ± S.E.M. A value of *P* < 0.05 was considered statistically significant.

## Additional Information

**How to cite this article**: Ding, L. *et al*. Reduced lipolysis response to adipose afferent reflex involved in impaired activation of adrenoceptor-cAMP-PKA-hormone sensitive lipase pathway in obesity. *Sci. Rep.* **6**, 34374; doi: 10.1038/srep34374 (2016).

## Supplementary Material

Supplementary Information
