# Supplementary material for: Reduced lipolysis response to adipose afferent reflex involved in impaired activation of adrenoceptor-cAMP-PKA-hormone sensitive lipase pathway in obesity
Source: Sci Rep. 2016 Oct 3;6:34374. doi: 10.1038/srep34374 (PMC5046068; doi:10.1038/srep34374)
Supplement: Supplementary Information [file srep34374-s1.doc]

**Reduced lipolysis response to adipose afferent reflex involved in impaired activation of adrenoceptor-cAMP-hormone sensitive lipase pathway in obesity**

Lei Ding^a^, Feng Zhang^a^, Ming-Xia Zhao^a^, Xing-Sheng Ren^a^, Qi Chen^b^, Yue-Hua Li^b^, Yu-Ming Kang^c^, Guo-Qing Zhu^a^,*****

^a^Key Laboratory of Cardiovascular Disease and Molecular Intervention, Department of Physiology, Nanjing Medical University, Nanjing, Jiangsu 210029, China; ^b^Department of Pathophysiology, Nanjing Medical University, Nanjing, Jiangsu 210029, China; ^c^Department of Physiology and Pathophysiology, Cardiovascular Research Center, Xi'an Jiaotong University School of Medicine, Xi'an 710061, China

***Address for correspondence:**

Guo-Qing Zhu, M.D., Ph.D. Professor, Chair

Key Laboratory of Cardiovascular Disease and Molecular Intervention, Department of Physiology, Nanjing Medical University, 140 Hanzhong Road, Nanjing 210029, China

Tel: +86-25-86862885

Fax: +86-25-86862885

E-Mail: [gqzhucn@njmu.edu.cn](mailto:gqzhucn@njmu.edu.cn)

**Supplemental Figures**

**
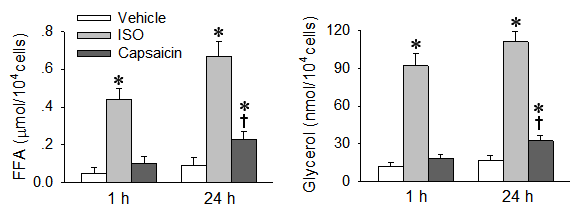
**

**Figure S1. Effects of ISO and capsaicin on FFA and glycerol release in primary SAT adipocytes derived from normal rats.** The adipocytes were treated with vehicle, isoproterenol (ISO, a β-adrenergic receptor agonist, 1 μM) or capsaicin (10 μM) for 1 h or 24 h. Values are mean±S.E.M. *P<0.05 vs. Vehicle; †P<0.05 vs. ISO. n=6 for each group.


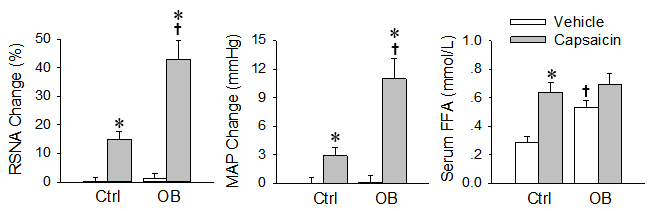


**Figure S2 Effects of injection of capsaicin into the pWAT on RSNA, MAP and FFA release in Ctrl and OB rats.** Values are mean±S.E.M. *P<0.05 vs. Vehicle; †P<0.05 vs. Ctrl rats. n=6 for each group.


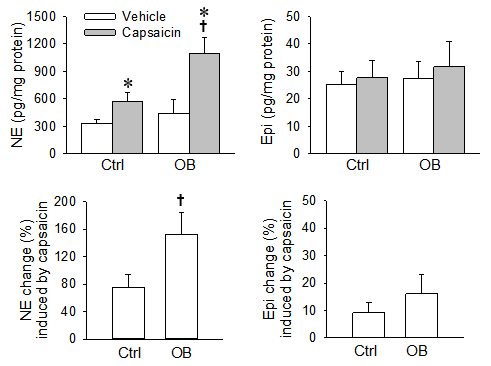


**Figure S3. Effects of capsaicin-induced AAR on norepinephrine (NE) and epinephrine (Epi) levels in adipose tissues of Ctrl and OB rats.** Values are mean±S.E.M. *P<0.05 vs. Vehicle; †P<0.05 vs. Ctrl rats. n=6 for each group.


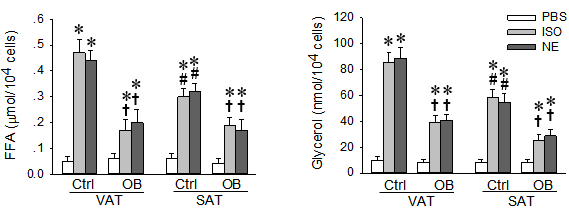


**Figure S4. Lipolysis effect of isoproterenol (ISO) and norepinephrine (NE) in adipocytes derived from Ctrl and OB rats.** Values are mean±S.E.M. *P<0.05 vs. PBS. †P<0.05 vs. Ctrl rats. #P<0.05 vs. VAT. n=6 for each group.

**Table S1 Anatomic data, MAP and HR**

| Variables | Ctrl | OB |
| --- | --- | --- |
| n | 30 | 43 |
| Initial body weight (BW), g | 316.6±2.1 | 314.1±2.7 |
| Final BW, g | 471.6±7.3 | 639.3±9.0* |
| BW gain, g | 155.1 ±7.9 | 325 ±9.9* |
| Final BW range, g | 370-549 | 557-813 |
| MAP, mm Hg | 90.1±1.1 | 113.4±3* |
| HR, bpm | 338.2±4 | 372.4±4.8* |

Values are mean±SE. * P<0.05 vs. Ctrl. MAP and HR were determined under anesthesia.

**Table S2** Primers for Real-time quantitative PCR analysis in rats

|  | Primer | Sequence | Accession number |
| --- | --- | --- | --- |
| HSL | Forward | 5'- CCGTTCCTGCAGACTCTCTC -3' | XM_008758896.1 |
|  | Reverse | 5'- CCACGCAACTCTGGGTCTAT -3' |  |
| β_1_-receptor | Forward | 5'-CTGCTACAACGACCCCAAGTG-3' | NM_012701.1 |
|  | Reverse | 5'-AACACCCGGAGGTACACGAA-3' |  |
| β_2_- receptor | Forward | 5'-GAGCCACACGGGAATGACA-3' | NM_012492.2 |
|  | Reverse | 5'-CCAGGACGATAACCGACATGA-3' |  |
| β_3_- receptor | Forward | 5'-TCTGTGTAACTGCCAGCATCGA-3' | NM_013108.2 |
|  | Reverse | 5'-TGGTAACCAGCGTGCCGTAA-3' |  |
| β-actin | Forward | 5'-ATGTGGATCAGCAAGCAGGA-3' | NM_031144.3 |
|  | Reverse | 5'-AAGGGTGTAAAACGCAGCTCA-3' |  |
